# Colour Parameters and Changes of Tea-Stained Resin Composite Exposed to Whitening Pen (In Vitro Study)

**DOI:** 10.3390/polym15143068

**Published:** 2023-07-17

**Authors:** Abdulaziz Alhotan, Rasha M. Abdelraouf, Saleh Alhijji, Merry Angelyn Tan De Vera, Aref Sufyan, Jukka P. Matinlinna, Tamer M. Hamdy

**Affiliations:** 1 Department of Dental Health, College of Applied Medical Sciences, King Saud University, P.O. Box 10219, Riyadh 12372, Saudi Arabia; aalhotan@ksu.edu.sa (A.A.); smalhijji@ksu.edu.sa (S.A.); 2 Biomaterials Department, Faculty of Dentistry, Cairo University, Cairo 11553, Egypt; rasha.abdelraouf@dentistry.cu.edu.eg; 3 College of Dentistry, King Saud University, P.O. Box 60169, Riyadh 11545, Saudi Arabia; mtandevera@ksu.edu.sa (M.A.T.D.V.); asufyan@ksu.edu.sa (A.S.); 4 Division of Dentistry, Faculty of Biology, Medicine and Health, The University of Manchester, Manchester M13 9PL, UK; jukka.matinlinna@manchester.ac.uk; 5 Restorative and Dental Materials Department, Oral and Dental Research Institute, National Research Centre (NRC), El Bohouth St., Dokki, Giza 12622, Egypt

**Keywords:** whitening pen, colour changes, tea staining, resin composite, Vita Easy Shade

## Abstract

Background: One of the crucial factors influencing the longevity of anterior aesthetic dental restorations is the colour stability of resin composites. Bleaching and whitening have become popular methods for enhancing dental aesthetics. Home whitening techniques, such as special pens, are widely available commercially. This in vitro study aimed to determine the efficiency of a whitening pen in removing tea stains from resin composite by measuring colour differences (ΔE_00_). Additionally, the study aimed to evaluate the variations in colour parameters measured by extra-oral and intra-oral spectrophotometers. Methods: A total of 45 disc-shaped resin composite specimens were randomly divided into three groups; Group 1: stored in artificial saliva (control), Group 2: stored in artificial saliva followed by a whitening pen application, and Group 3: stored in tea followed by a whitening pen application. Colour measurements were taken three times for each specimen using two spectrophotometers (extra-oral and intra-oral devices); T1: before storage (baseline), T2: after storage in artificial saliva or tea for 6 days; and T3: after one week of whitening pen application in groups 2 and 3. The data were statistically analyzed using one-way ANOVA followed by the Tukey post hoc test (*p* ≤ 0.05). The independent sample *t*-test was also employed. The equation of CIEDE2000 (ΔE_00_) was used to calculate the colour difference between the dry, as-prepared specimens (baseline), and those after storage or bleaching. The colour changes exceeding the acceptability threshold (∆E_00_ = 1.8) were considered unacceptable. Results: After whitening, the colour of the specimens stored in brewed tea (Group 3) remained unacceptable, as indicated by both the extra-oral and intra-oral spectrophotometers (ΔE_00_ = 4 and 2.9, respectively). Groups 1 and 2 exhibited lower ΔE_00_ values than Group 3 (*p* = 0.01 *). No significant difference was observed between Group 1 (stored in artificial saliva) and Group 2 (stored in artificial saliva and then bleached) (*p* = 0.3). Significant differences were consistently observed between the data obtained from the extra-oral spectrophotometer and the intra-oral one. Conclusions: The whitening pen proved ineffective in removing tea stains from resin composites. Although significant differences were found between the values obtained by the two spectrophotometers (extra-oral and intra-oral), both devices confirmed the unacceptable colour of the tea-stained resin composites after whitening.

## 1. Introduction

Aesthetics is a significant concern for patients seeking dental treatment. Resin composite is a commonly used direct restorative material, and achieving a proper colour match with the adjacent teeth is crucial initially and over time [1,2,3]. However, frequent exposure of resin composite to beverages can result in discolouration [4,5].

Intrinsic or extrinsic factors can cause discolouration of intra-oral resin composite restorations. Intrinsic factors are related to the composition of the resin composite, such as the type of photo-initiator and the resin matrix. On the other hand, extrinsic factors involve the adsorption or absorption of stains from external sources primarily influenced by an individual’s diet and habits. Tea and coffee, for example, are common beverages that discolour teeth and tooth-coloured restorations, posing aesthetic and frustrating problems for patients [6,7]. This prevalent issue has prompted several companies to introduce dental whitening products, such as whitening pens, which typically rely on hydrogen peroxide (H_2_O_2_). However, the efficiency of whitening pens in removing stains from resin composite has not been widely investigated and addressed.

Two whitening methods that have been discussed are: (1) in-office, performed by a dental professional; this method uses photo-activation and stimulates colour changes from the first session; and (2) at-home (over-the-counter) [8]. At-home whitening is more cost-effective for the patient than the in-office approach [4]. However, significant changes will not be noticed until the seventh day of the treatment. Home whitening can be performed using intraoral trays. Several companies have launched at-home whitening methods, such as whitening pens [9].

In general, the procedures for dental bleaching and whitening rely on applying hydrogen peroxide or its precursor, carbamide peroxide (which breaks down into hydrogen peroxide) [10]. The hydrogen peroxide produces highly reactive species, free radicals, transforming the pigmented organic chromophores into non-coloured molecules. In-office whitening uses 35–40% hydrogen peroxide, and the patient should be exposed to the peroxide application for 15–20 min [11]. However, there is a risk of post-operative sensitivity. Home whitening agents contain a lower hydrogen peroxide content with lower resultant sensitivity, e.g., the whitening pen contains 3% hydrogen peroxide [12,13,14]. The available literature is not widely reporting the efficiency of whitening pens in removing stains. This is a specific lack of understanding of the effect of such pens on resin composite restorations, which are prone to discolouration due to their polymeric organic matrix, which affects their colour stability.

To assess the colour stability of the dental resin composite, a spectrophotometric shade has been recognized as an accurate and reliable tool designed for both extra-oral and intra-oral use. Extra-oral spectrophotometers are considered the gold standard, while intra-oral spectrophotometers have been developed to facilitate colour determination [5]. Data obtained from these devices are used to calculate the colour change using the CIEDE2000 colour difference (∆E_00_), which overcomes the weak points in the old E formula of the L*a*b*. The resultant ∆E_00_ values are compared to the perceptibility threshold, which measures the degree of colour variation seen visually [15,16]. The CIEDE 2000 has determined this to be 0.8. When the acceptability threshold is 1.8, the colour difference’s magnitude denotes an aesthetically unsuitable upper limit [15].

The objectives of this in vitro study were to (1) evaluate the efficiency of a whitening pen in removing stains from resin composites caused by tea, measured through colour differences (ΔE), and (2) compare colour parameters obtained from an intra-oral spectrophotometer with those from an extra-oral spectrophotometer. The null hypothesis stated that there would be no difference in colour before and after bleaching using the whitening pen, as assessed by the two spectrophotometers.

## 2. Materials and Methods

The present experimental study was approved by the Medical Research Ethical Committee (MREC) of National Research Centre (NRC), Cairo, Egypt (Reference number: 2430122022). Materials used in the study are presented in Table 1.

### 2.1. Study Design

**Figure 1:**
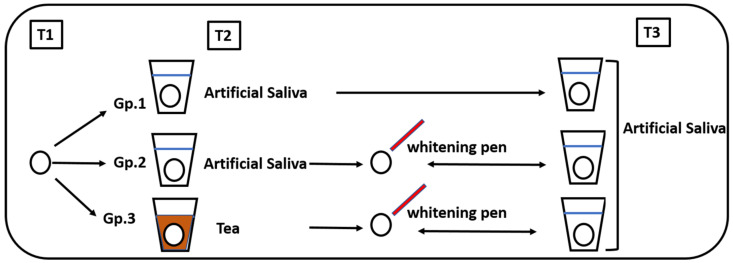
Diagram showing the study design.

A total of 45 disc-shaped resin composite specimens were randomly divided into 3 groups based on the storage media application of the whitening pen (Figure 1):

- Group 1: Stored in artificial saliva (control)
- Group 2: Stored in artificial saliva followed by whitening pen application
- Group 3: Stored in tea followed by whitening pen application

For each specimen, the colour was measured three times at intervals:

- T1: Before storage (baseline)
- T2: After storage in either artificial saliva or tea for 6 days
- T3: After the whitening pen application for one week in groups 2 and 3

The colour of each specimen was measured using both an extra-oral spectrophotometer and an intra-oral spectrophotometer.

### 2.2. Sample Size Calculation

The sample size was calculated using the G*Power (version 3.1.9.7) sample size calculator based on means and standard deviations [17,18]. The estimated sample size was 15 per group.

### 2.3. Specimen Preparation

A Teflon mound (8 mm in diameter and 1 mm in thickness) was used to prepare the disc-shaped specimens [2]. The mould was first filled with resin composite, covered with a translucent celluloid strip (Mylar strip; SS White, Philadelphia, PA, USA), and then a glass slide (1 mm thickness) was placed on top. Light, gentle pressure was applied to remove excess material and create a polished surface. A light emitting diode (LED) curing unit (Mini-LED, Satelec, Acteon, France) with a wavelength of 400–500 nm and a power density of 1000 mW/cm^2^ was used to cure the specimens for 40 s, following the manufacturer’s instructions.

### 2.4. Specimen Grouping, Storage, and Bleaching

- Group 1: specimens were incubated in 10 mL of artificial saliva (Sigma-Aldrich CO, St. Louis, MO, USA) at 37 °C for 11 days.
- Group 2: specimens were incubated in 10 mL of artificial saliva at 37 °C for 6 days (simulating 6 months intra-orally) [19,20] and then subjected to the whitening pen for one week (7 days).
- Group 3: specimens were incubated in 10 mL of tea at 37 °C for 6 days and then subjected to a whitening pen application for 7 days. The tea was prepared by steeping one tea bag (Lipton Yellow Label, Unilever, UK) for 5 min in 200 mL of boiling water.

Whitening with the pen was performed according to the manufacturer’s instructions for Groups 2 and 3. The tip of the whitening pen was applied to the surface of each specimen and allowed to dry for 60 s, forming a visible coat. The coated specimens were then immersed in artificial saliva for 8 h, simulating overnight exposure. Afterward, the coat was peeled from the specimen’s surface, simulating the removal of the coat from the teeth in the morning. The uncoated specimens were re-immersed in artificial saliva for the remaining 16 h of the day. This procedure was repeated for one week.

### 2.5. Colour Measurement

The colour parameters of each specimen were measured as the baseline (T1) using both an extra-oral spectrophotometer (Cary 5000, Agilent Technologies, Santa Clara, CA, USA) and an intra-oral spectrophotometer (Vita Easy Shade, VITA Zahnfabrik, Bad Säckingen, Germany) against a black background. After immersing specimens in artificial saliva (Groups 1 and 2) or tea (Group 3) for 6 days, the colour was re-measured (T2). The colour was re-assessed after the whitening process and/or storage for 1 week (T3).

The colour difference (∆E_00_) was calculated using the CIEDE2000 (La Commission Internationale de l’Éclairage) L*a*b* system with the CIE standard illuminant D65. The calculation employed the following formula colour [15,16,21]:

(1) $$\Delta E_{00}=\sqrt{{\left(\frac{\Delta L\prime }{k_{L}\cdot S_{L}}\right)}^{2}+{\left(\frac{\Delta C\prime }{k_{c}\cdot S_{c}}\right)}^{2}+{\left(\frac{\Delta H\prime }{k_{H}\cdot S_{H}}\right)}^{2}+\left(R_{T}\left(\frac{\Delta C\prime }{k_{C}\cdot S_{C}}\right)\left(\frac{\Delta H\prime }{k_{H}\cdot S_{H}}\right)\right)}$$

where ΔL = the lightness difference, ΔC = the chroma difference, ΔH = the hue difference, and k_L_, S_L_, k_C_, S_C_, k_H_, and S_H_ are the constants of the coefficient.

The resultant ∆E_00_ values were compared to the perceptibility threshold, which represents the initial visually detectable colour variation (∆E_00_ = 0.8), and the acceptability threshold, which represents the starting point of unacceptable colour change (∆E_00_ = 1.8) [11].

### 2.6. Statistical Analyses

The data were statistically evaluated using the Statistical Package for the Social Sciences, version 26 for Windows (SPSS, IBM, New York, NY, USA). Mean and standard deviation were used to describe the data, which showed a normal distribution using the Shapiro-Wilk and Kolmogrov-Smirnov tests. A significance level of α ≤ 0.05 was considered. When comparing three groups, mean colour differences (ΔE_00_) and colour parameters were compared using One-way ANOVA followed by the Tukey post hoc test. When comparing two groups, an independent sample *t*-test was used.

## 3. Results

**Figure 2:**
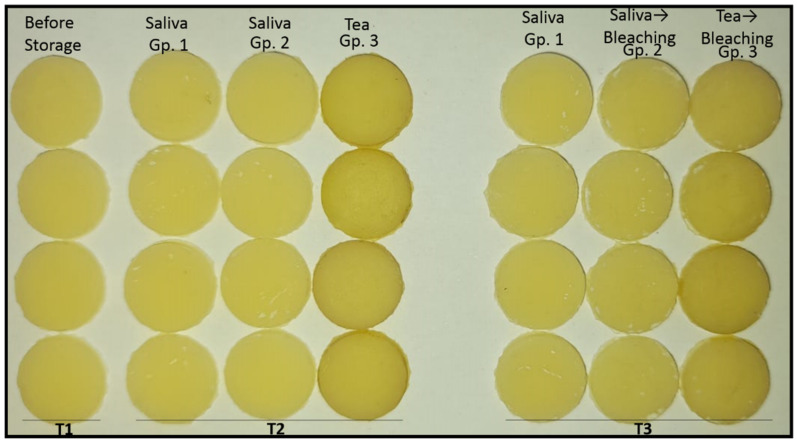
Representative specimens from the different study groups.

Figure 2 represents the colour of the different groups at the three time measurement points.

The specimens stored in tea (Group 3) exhibited significantly higher colour changes compared to those stored in artificial saliva (Groups 1 and 2), with significant differences in the ΔE_00_ values (*p* = 0.0001 *), as shown in Table 2. Tea resulted in unacceptable colour changes, as detected by both extra-oral and intra-oral spectrophotometers (ΔE_00_ = 5.8 and 3.7, respectively), exceeding the acceptable threshold value (ΔE_00_ = 1.8). On the other hand, the colour changes observed in the artificial saliva groups were acceptable, with ΔE_00_ values exceeding the perceptibility threshold (ΔE_00_ = 0.8) when detected by the extra-oral spectrophotometers (ΔE_00_ = 1), but below the acceptability threshold when detected by intra-oral spectrophotometers (ΔE_00_ = 0.4). Significant differences in ΔE_00_ values were found between both spectrophotometers in all groups (*p* = 0.0001*).

After whitening, the colour of the specimens stored in tea (Group 3) remained unacceptable, as detected by both extra-oral and intra-oral spectrophotometers (ΔE_00_ = 4 and 2.9, respectively), as shown in Table 3. Groups 1 and 2 showed lower ΔE_00_ values than Group 3 (***p*** = 0.01 *). No significant difference was found between Group 1 (stored in artificial saliva) and Group 2 (stored in artificial saliva and then bleached), *p* = 0.3.

When detected by extra-oral spectrophotometers, the colour change in Groups 1 and 2 was considered unacceptable (ΔE_00_ = 2.8), whereas, when detected by the intra-oral spectrophotometers, the colour changes were acceptable but still exceeded the perceptibility threshold (ΔE_00_ = 1.3 and 1.5. in Groups 1 and 2, respectively).

Significant differences were observed in the colour parameters (L, a, and b) detected by extra-oral and intra-oral spectrophotometers for the specimens before storage (T1), as shown in Table 4. The colour parameters measured by the extra-oral spectrophotometer were lower than those recorded by the intra-oral spectrophotometer. The mean L value measured by the extra-oral spectrophotometer (L = 68.1) was significantly lower than that detected by the intra-oral one (L = 93.9). Similarly, the ‘b’ values measured by the extra and intra-oral spectrophotometers were 5.1 and 39.5, respectively. The mean ‘a’ values measured by the extra-oral and intra-oral spectrophotometers were −1.8 and −1.1, respectively.

Table 5 presents the colour parameters (L, a, and b) after storage (T2) in artificial saliva (Groups 1 and 2) and tea (Group 3). Compared to storage in artificial saliva, the tea showed a decrease in the L value of the specimens, indicating increased darkness, and an increase in a (+a = red) and b values (+b = yellow), indicating increased redness and yellowish tint. Both spectrophotometers detected these findings, although significant differences existed in the numerical values obtained by the two devices.

Table 6 displays the colour parameters (L, a, and b) after whitening (T3). Similar to the phase, the specimens stored in tea still exhibited a decreased L value, indicating increased darkness, and an increase in a (+a = red) and b values (+b = yellow), indicating increased redness and yellowish tint. Both spectrophotometers detected these changes, with significant differences in the numerical values obtained.

**Figure 3:**
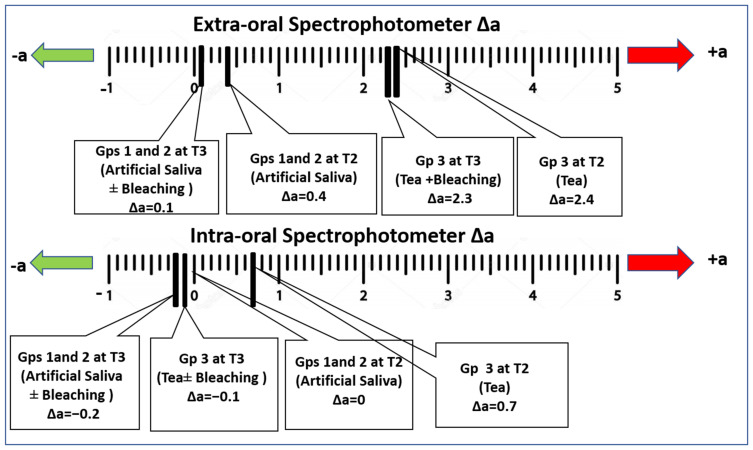
Changes in the hue across the red–green axis (Δa) compared to the baseline colour (T1).

Figure 3 illustrates the changes in hue of the specimens along the red-green axis (Δa) at T2 and T3, compared to the baseline colour (T1). Generally, after whitening (T3), there was a decrease in the Δa value compared to T2 (↓+a = ↓red), indicating decreased redness within the same group. These findings were detected by both spectrophotometers, with significant differences in the numerical values obtained.

**Figure 4:**
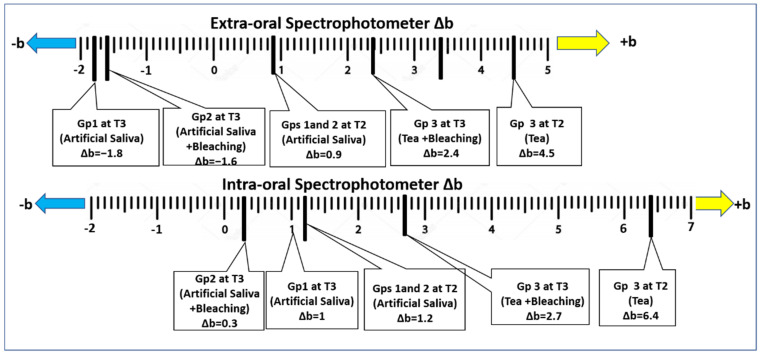
Changes in the hue across the yellow–blue axis (Δb) compared to the baseline colour (T1).

Figure 4 displays the changes in the hue of the specimens along the yellow-blue axis (Δb) at T2 and T3, compared to the baseline colour (T1). Generally, after whitening (T3), there was a decrease in the Δb value compared to T2 (↓+b = ↓yellow), indicating decreased yellowish discolouration within the same group. Both spectrophotometers detected these changes, with significant differences in the numerical values obtained.

**Figure 5:**
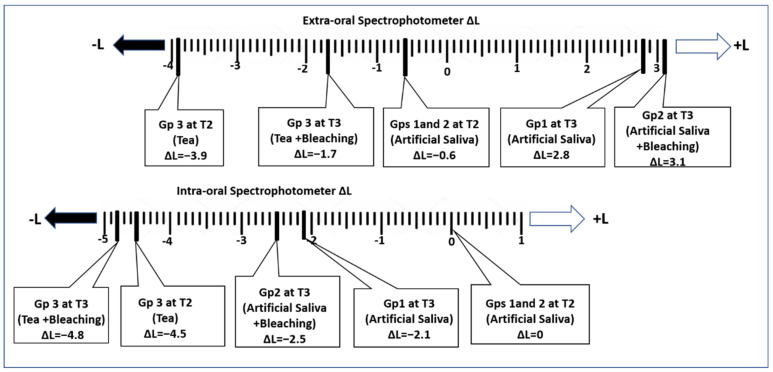
Changes in the lightness or darkness (ΔL) compared to the baseline colour (T1).

Figure 5 demonstrates the changes in the degree of greyness (ΔL) of the specimens at T2 and T3, compared to the baseline colour (T1). The extra-oral spectrophotometer showed an increased ΔL value after whitening (T3) compared to T2 (↑+L = ↑Lighter), indicating a lighter colour within the same group. Conversely, the intra-oral spectrophotometer showed a decreased L value after whitening, indicating a darker colour.

## 4. Discussion

Several polymeric materials are commonly utilized in dentistry [22,23,24,25,26], where colour matching and stability are crucial for an aesthetic appearance [27,28]. The surface characteristics of resin composites can be affected by various beverages, including tea, which has been a reported cause of discolouration of teeth and restorations [29]. With the availability of home-based whitening techniques in the dental market, such as whitening pens, it is important to investigate their efficiency in stain removal, as this aspect has not yet been widely studied.

In the current laboratory study, specimens were stored at 37 °C to simulate oral temperature for 6 days, representing a six-month clinical period [30]. The control group consisted of specimens incubated in artificial saliva, while the second group included specimens incubated in artificial saliva at 37 °C and subjected to whitening. There is controversial literature regarding the effect of whitening agents on resin composites, with some studies reporting a whitening effect and others reporting no colour change. The third group involved specimens incubated in tea at 37 °C and subjected to the application of a whitening pen.

The colour parameters of each specimen were measured at three time points: T1 (baseline before immersion), T2 (after immersion in artificial saliva or tea), and T3 (after whitening and/or storage). The colour assessment was performed using two spectrophotometers: an extra-oral device and an intra-oral device. In dental research, intra-oral devices such as Vita™ Easyshade have been commonly used for assessing clinical cases [16,31,32]. Therefore, analyzing the colour parameters and differences and comparing the obtained data to the standard extra-oral spectrophotometers could be beneficial [16,31].

In this research, a slight colour change was observed in the specimens stored in the artificial saliva for 6 days (T2), as detected by both the extra-oral and intra-oral spectrophotometers, ΔE_00_ = 1 and 0.4, respectively. This colour change may be attributed to water sorption occurring within the resinous part of the composite. Two main models have been proposed to explain the water uptake in the polymeric matrices: the “free volume theory”, where water penetrates through the nanopores without interacting chemically with the polymeric chains, and according to the “interaction theory,” water interacts with the hydrophilic groups [33].

On the other hand, the specimens stored in tea for 6 days resulted in unacceptable colour changes in the resin composite, as detected by the extra-oral and intra-oral spectrophotometers (ΔE_00_ = 5.8 and 3.7, respectively). This can be attributed to the high staining effect of the colouring ingredients of tea, which has been verified in previous studies [34,35]. In this research, the colour change in the tea-stained specimens was analyzed by assessing the colour parameters, which showed a decrease in the L value, indicating increased darkness and an increase in the a (+a = red) and b values (+b = yellow) indicating increased redness and a yellowish hue, compared to the specimens stored in artificial saliva.

After the whitening process (T3), the specimens stored in tea showed an improvement in colour as evidenced by a reduction in the colour difference values compared to before whitening, ΔE_00_ = 4 and 2.9, detected by extra-oral and intra-oral spectrophotometers, respectively. This improvement could be attributed to the 3% hydrogen peroxide in the whitening pen. The whitening compound, H_2_O_2_, dissociates into free radicals, which reduce large pigmented molecules into smaller, less-pigmented entities [36,37].

However, the colour of the specimens stored in tea was still unacceptable, exceeding the acceptability threshold (ΔE_00_ = 1.8), with a decreased L value indicating increased darkness and an increase in a and b values representing increased redness and a yellowish hue compared to the control specimens. This may be due to the superficial effect of the whitening pen, which forms a coat on the surface of the specimens upon drying. On the other hand, the pigment molecules in tea can penetrate deep into the polymeric restorative materials, causing bulk discolouration, as reported previously [38]. Since there was no effective difference in colour before and after whitening when using the two spectrophotometers, the authors failed to reject the null hypothesis.

Resin composite specimens stored in artificial saliva and then whitened (Group 2) showed no significant difference compared to the specimens stored in artificial saliva alone (group 1) at T3 (*p* = 0.3). This indicates that whitening had no pronounced effect on the colour of the unstained resin composite. Thus, the colour change observed increased in Groups 1 and 2 at T3 was primarily caused by storage rather than whitening, which might be attributed to an increased water uptake over time. When assessed by the extra-oral spectrophotometer, the resultant colour changes in Groups 1 and 2 at T3 were considered unacceptable (ΔE_00_ = 2.8). However, they were considered acceptable when detected by the intra-oral spectrophotometer (ΔE_00_ = 1.3 and 1.5 in Groups 1 and 2, respectively). This discrepancy might be due to the different calibrations of the two devices, as confirmed by the significant differences in the observed differences in the colour parameters (L, a, and b) between the extra-oral and intra-oral spectrophotometers. The colour parameters measured by the extra-oral spectrophotometer were observed to be lower than those measured by the intra-oral device. This variation could be attributed to calibration and settings between the two devices. Therefore, confirming the results with a visual assessment as an adjunctive method to instrumental calculation might be beneficial. Additionally, standardization of the colour parameter settings is highly recommended among different brands of spectrophotometers, especially when comparing the resultant colour difference (ΔE_00_) to specific values determined in the literature for perceptibility and acceptability thresholds. Researchers should compare the ΔE_00_ values resulting from their experiments to these values, regardless of the type of the colour measuring device. Th variation among devices can lead to confusion, as observed when comparing the ΔL values for the specimens at T3 vs. T2. The extra-oral spectrophotometer showed an increased ΔL value after whitening (T3) compared to T2 (↑+L = ↑Lighter), indicating a lighter colour within the same group. On the other hand, the intra-oral spectrophotometer showed a decreased L value after whitening, indicating a darker colour. Therefore, standard settings of the colour parameters for colour measuring tools will lead to more accurate data describing the real situation.

One limitation of this study was the use of only one type of resin composite, and the application of the whitening pen was limited to one week. It is recommended to compare the effect of the whitening pen on different types of resin composites subjected to various beverages for longer durations and to compare it to other whitening techniques. Further studies on extracted teeth and clinical cases are recommended.

## 5. Conclusions

The whitening pen was ineffective in removing tea stains from the resin composite. Despite significant differences in the values obtained by the two spectrophotometers (extra-oral versus intra-oral), both devices indicated the unacceptable colour of the tea-stained resin composite after bleaching. Therefore, standardizing the colour measuring devices to ensure more accurate data is recommended.

## Figures and Tables

**Table 1:** Materials used in the study.

Material	Commercial Product	Composition
Resin composite	Luna (Anterior/Posterior Nano- Hybrid Composite, Shade A2), SDI, Victoria, Australia	22.5 wt.% (39 vol.%) multifunctional methacrylic ester, 77.5 wt.% (61 vol.%) inorganic fillers (40 nm–1.5 μm).
Whitening pen	Colgate Optic White Overnight Teeth Whitening Pen, Colgate, NY, USA	3% Hydrogen peroxide, acrylates/octylacrylamide copolymer, alcohol, water.

**Table 2:** ΔE_00_ before and after storage for 6 days in artificial saliva (Group 1 and 2) and in tea (Group 3): The difference between T1 and T2.

Device	Group 1 and Group 2 (Artificial Saliva)	Group 3 (Tea)	*p*-Value
Extra-oral spectrophotometer	1.0 ± 0.1	5.8 ± 0.04	*p* = 0.0001 *
Intra-oral spectrophotometer	0.4 ± 0.2	3.7 ± 0.5	
*p*-value	*p* = 0.0001 *	*p* = 0.0001 *	*p* = 0.0001 *

* Significantly different (*p* ≤ 0.05).

**Table 3:** ΔE_00_ for the specimens before storage and after whitening (difference between T1 and T3).

Device	Group 1 (Artificial Saliva)	Group 2 (Saliva → Whitening)	Group 3 (Tea → Whitening)	*p*-Value
Extra-oral Spectrophotometer	2.8 a ± 0.1	2.8 a ± 0.1	4 b ± 0.3	*p* = 0.01 *
Intra-oral Spectrophotometer	1.3 a ± 0.2	1.5 a ± 0.3	2.9 b ± 0.4	
*p*-value	*p* = 0.0001 *	*p* = 0.0001 *	*p* = 0.0001 *	*p* = 0.001 *

Mean with different letters indicate statistically significant difference, * Significantly different (*p* ≤ 0.05).

**Table 4:** Colour parameters (L, a, and b) for the specimens before storage (T1).

Device	L	a	b
Extra-oral Spectrophotometer	68.1 ± 0.2	−1.8 ± 0.06	5.1 ± 0.2
Intra-oral Spectrophotometer	93.9 ± 0.4	−1.1 ± 0.1	39.8 ± 0.2
*p*-value	*p* = 0.0001 *	*p* = 0.003 *	*p* = 0.0001 *

* Significantly different (*p* ≤ 0.05).

**Table 5:** Colour parameters (L, a, and b) after storage (T2) in artificial saliva (Groups 1 and 2) and tea (Group 3).

Device	L Artificial Saliva	L Tea	a Artificial Saliva	a Tea	b Artificial Saliva	b Tea
Extra-oral Spectrophotometer	67.5 ± 0.1	64.2 ± 0.1	−1.4 ± 0.1	0.6 ± 0.08	6.0 ± 0.1	9.6 ± 0.1
Intra-oral Spectrophotometer	93.9 ± 0.4	89.4 ± 0.4	−1.1 ± 0.1	−0.4 ± 0.2	41.0 ± 0.5	46.2 ± 1.4
*p*-value	*p* = 0.0001 *	*p* = 0.0001 *	*p* = 0.003 *	*p* = 0.003 *	*p* = 0.0001 *	*p* = 0.0001 *

* Significantly different (*p* ≤ 0.05).

**Table 6:** Colour parameters (L, a, and b) after bleaching (T3).

Device	L Group 1	L Group 2	L Group 3	a Group 1	a Group 2	a Group 3	b Group 1	b Group 2	b Group 3
Extra-oral Spectrophotometer	70.9 ± 0.1	71.2 ± 0.1	66.4 ± 0.10	−1. 7± 0.1	−1.7 ± 0.1	0.5 ± 0.1	3.3 ± 0.1	3.5 ± 0.1	7.5 ± 0.1
Intra-oral Spectrophotometer	91.8 ± 1.0	91.4 ± 0.6	89.1 ± 1.5	−1.3 ± 0.3	−1.3 ± 0.2	−1.2 ± 0.2	40.8 ± 0.5	40.1 ± 0.5	42.5 ± 0.9
*p* value	*p* = 0.0001 *	*p* = 0.0001 *	*p* = 0.0001 *	*p* = 0.0001 *	*p* = 0.0001 *	*p* = 0.0001 *	*p* = 0.0001 *	*p* = 0.0001 *	*p* = 0.0001 *

* Significantly different (*p* ≤ 0.05).

## Data Availability

The data presented in this study are available on request from the corresponding author.
